# Pattern of comorbidities in school-aged children with cerebral palsy in Cross River State, Nigeria

**DOI:** 10.1186/s12887-021-02637-9

**Published:** 2021-04-08

**Authors:** Roseline E. Duke, Chimaeze Torty, Uche Okorie, Min J. Kim, Nnena Eneli, Ukam Edadi, Kathryn Burton, Cally Tann, Richard Bowman

**Affiliations:** 1 London School of Hygiene & Tropical Medicine, International Centre for Eye Health, London, UK; 2 Department of Ophthalmology, University of Calabar Teaching Hospital, Calabar Children’s Eye Centre, Calabar, Cross River State Nigeria; 3 Department of Paediatrics, Neurology Unit, University of Calabar Teaching Hospital, Calabar, Nigeria; 4 Department of Physiotherapy, University of Calabar Teaching Hospital, Calabar, Nigeria; 5 Primary Health Care Development Agency, Calabar, Cross River State Nigeria; 6 Cambridgeshire Community Services, Cambridge, UK; 7 Clinical Research Department, London School of Hygiene & Tropical Medicine, London, UK

**Keywords:** Cerebral palsy, Comorbidity, School attendance, Children

## Abstract

**Background:**

To describe the pattern of comorbidities in school-aged children with cerebral palsy (CP) and to identify which, if any, were associated with poor school attendance.

A cross-sectional study, using the key informant methodology, between December 2017 and July 2018 was conducted in Cross River State, Nigeria. Assessments, confirmation of CP and identification of systemic comorbidities using standard tools and questionnaires were performed. Children confirmed to have CP between the ages 4 to 15 years were included.

**Results:**

Three hundred and eighty-eight children were confirmed to have CP, 59% males. The mean age was 9.2 years ± SD 4.0; 28% were non-ambulatory (gross motor function classification system (GMFCS) level IV-V) and spastic CP was seen in 70%. Comorbidities included Speech impairment 85%, feeding difficulties 86%, and swallowing difficulties 77%, learning difficulties 88%, abnormal behaviour 62%, visual acuity impairment 54%, objective perceptual visual disorders 46%, communication difficulties 45%, epilepsy 35%, hearing impairment 12% and malnutrition 51%. Learning difficulties (OR 10.1, *p* < 0.001; CI: 3.6–28.1), visual acuity impairment (OR 2.8, *p* = 0.002; CI: 1.5–5.3), epilepsy (OR 2.3, *p* = 0.009; CI:1.2–4.3) manual ability classification scale 4–5 (OR 4.7,*p* = 0.049; CI:1.0–22.2) and CP severity (GMFCS V-VI) OR 6.9 *p* = 0.002, CI: 2.0–24.0.) were seen as increasing the likelihood of poor school attendance.

**Conclusion:**

Comorbidities were common, and some were associated with limited school attendance. A multidisciplinary tailored approach to care, with application of available therapeutic interventions for comorbidities is suggested. This may be useful in reducing barriers to school attendance.

**Supplementary Information:**

The online version contains supplementary material available at 10.1186/s12887-021-02637-9.

## Introduction

Cerebral palsy (CP) is a leading cause of childhood disability across the world with significant impact on function and development [1]. The definition of CP highlights frequent association with disorders of sensation, perception, cognition, communication, behaviour, with epilepsy and secondary musculoskeletal problems [2]. Co-morbidities affect the overall health and quality of life in children by determining their participation in most aspects of life including schooling [3]. Secondary conditions (e.g. joint contractures and malnutrition) occur and are preventable. Children with more severe levels of gross motor dysfunction present with comorbidity more frequently [4, 5]. It is estimated that the majority of children with disabilities in Africa do not go to school at all [6], and of the 72 million primary aged children worldwide that are out of school, one third have disabilities [7]. Few reports describe school attendance and educational attainment in children with CP in Low and Middle Income Countries (LMIC).

Children with CP and disability in general face barriers and challenges to education [6]. Barriers to attending school are commonly described through the ‘social model’ of disability, that is about the way the society responds to children with disability. The common ones are physical, social and financial [8]. This ‘social model’ of disability differs from the ‘medical model’ which sees people with disabilities as having a problem that needs to be managed, changed and/or adapted to circumstances [9].

Co-morbidities may contribute to these barriers. Little evidence exists as to whether and which comorbidities are significant barriers to participation in schooling. Identification of these comorbidities may be important to attain effective individualized support measures for education.

In Nigeria, there are strong regional disparities in education and socioeconomic indices, with the southern region of the country (where Cross River State is situated) performing better in indices, including of health, education and the millennium development goals [10]. In the general population of children in Nigeria, the gross enrolment rate in elementary school is 68.3%, gross enrolment rate in lower secondary is 54.4% and upper secondary 68.9% [11] With regards to malnutrition, 37% of children are stunted and in addition 18% of children suffer from wasting while 29 % of children are underweight [12].

The aim of this study was to describe the comorbidities seen in children with CP in this community –based Nigerian population, and to identify which comorbidities were associated with poor school attendance.

## Methods

### Study setting/context

A population based cross sectional study was conducted in Cross River State (CRS) in Nigeria between December 2017 and July 2018 and has been reported in other articles [13–15].

Primary and secondary education enrolment is compulsory; however, it is paid for by the government for children in the primary school, while the government subsidizes the examination fees for the secondary education. Located in every village is a primary school and in every local government area there is a state government secondary school and a mission secondary school. Enrolment into kindergarten is from 3 years old. Secondary school education is divided into junior secondary and senior secondary schools [16]. There are three government special education schools in the state for deaf, mute and visually impaired children.

### Sample size

Existing data from this world region suggest a population prevalence of CP of 2.9 per1000 children and about the same estimate for associated comorbidities [17–19]. Considering the population size for the total number of children aged 4-15 years in Cross River State as 1.1 M [20], a 10% non-response rate, a sample size of 370 would be sufficient to estimate a prevalence of 2.9/1000 (i.e. *p* = 0.29%) and associated comorbidities [17] with a level of precision of ±0.58%.

### Sampling strategy

In the absence of a cerebral palsy registry, the key informant methodology (KIM) was selected as the most cost effective sampling strategy to identify children with CP and other disabilities in our circumstances. Several researches have validated this method [21–24]. The key informant methodology (KIM) was chosen, recognising its effectiveness for identifying physical impairment compared to household survey in LMIC [25]. The methodology used is referred to in other articles [13–15].

Identified and referred children were then assessed first of all by a paediatric neurologist in the primary health centre to determine if they met the inclusion criteria for CP. Families were provided with appropriate advice, information and counselling, referral services and intervention where appropriate. CP was defined according to history and neurological examination in line with international criteria [26]. Gross Motor Function Classification Scale (GMFCS) was used to describe the severity of CP of gross motor impairment. These levels were categorized into ambulatory (Levels I–III) and non-ambulatory (Levels IV–V) [27]. Validated existing questionnaires and tools were used such as the strength difficulties questionnaire, the manual ability classifications system and the communication function classification system.

The inclusion criteria included, children aged between 4 and 15 years of age at their last birthday, who were confirmed to have CP from history and clinical assessment by a paediatric neurologist [28]. Exclusion criteria included children who had other motor disorders apart from CP and children outside the age criteria and those that refused to participate in the study.

### Comorbidity case ascertainment

Comorbid conditions were confirmed by history, clinical and standardized evaluations. Comorbidities investigated included: epilepsy, hearing impairment, feeding difficulties, swallowing difficulties, visual acuity impairment, objective perceptual visual disorders, abnormal behaviour, learning difficulty, speech impairment, communication difficulties and malnutrition.

The Lea symbols cut-off point for screening preschool children of 0.8 was used as score for normal visual acuity and > 0.8 were considered to have visual acuity impairment [29]. Objective perceptual visual impairment was ascertained by a battery of tests [14].

Hearing impairment was assessed using three-level voice test (for children able to participate) and was present when a child failed to respond to mid-level spoken voice in either ear. Learning difficulty was assessed, by clinical history, assessment and behavioural observation [30, 31]. The Communication Function Classification Scale (CFCS) assessed the full activity of communication in five levels between a familiar person and the child [32]. We referred to children as having communication impairment if CFCS was level 4–5. Speech impairment were defined as inability to create or form speech sounds [33]. Epilepsy was diagnosed on a history of having two unprovoked seizures > 24 h apart at any time from 1 month of age to assessment [34]. Feeding difficulties was based on the reported ability of the child to chew food and the need for food to be cut up or mashed. Swallowing difficulties was defined as choking and coughing on food or drink based on parental report [35]. The manual ability classification system (MACS) [36], categorized as 1–3 and 4–5 as severe manual ability impairment. The strength and difficulties questionnaire(SDQ) [37], which is an emotional and behavioural screening questionnaire, was used through parent interview to describe any behavioural abnormality, emotional and conduct problems, hyperactivity, peer problems and prosocial behaviours [38]. Behaviour disorder was defined using the total difficulties score from the Strength and Difficulties Questionnaire. Malnutrition was classified using the Centre for Disease Control (CDC) growth chart (for ages 2–19 years) [39, 40], and comprised of underweight or wasting, stunting and overweight. Severe acute malnutrition was defined as weight for height at least 3 SD below the reference median or mid- upper arm circumference less than 11.5 cm for children less than 5 years [41].

Participation in mainstream schooling was recorded alongside whether children were at expected levels within the school programme by parent’s report. Dropout in school was defined as the percentage of students failing to complete a particular school year or school program.

### Statistical analysis

Statistical analyses were performed using Stata 15 (Stata Corp LP, College Station, TX). Descriptive statistics were reported using means, standard deviations, medians, interquartile ranges, frequencies and percentages. Comparisons between categorical variables were performed using a chi-square test and logistic regression.

Comorbidity score was calculated by summation of the frequencies of the following comorbidities: feeding, swallowing, hearing, speech, learning, visual acuity impairments and objective perceptual visual disorders. In addition to; malnutrition, communication difficulties, epilepsy and abnormal behaviours. This was followed by the calculation of the comorbidity mean.

The Kruskal Wallis test was used to determine the association of comorbidity scores with the type of CP. Significance level was set at *P* < 0.05. Missing data if less than 30% were included as normal. The influence of co-morbidities and CP severity on school attendance was assessed in a bivariate analysis. Correlations between factors predicting school attendance were the sought.

Multiple logistic regression models, adjusted for age and sex, were developed to identify factors associated with poor school attendance. Variables included in the regression models included systemic comorbidities and severity of CP. These were chosen based on biological plausibility and findings from previous studies. In addition, a no selection procedure was used to include other factors: variables significant at *p* < 0.2 level, or not significant at *p* > 0.2 but with an odds ratio between 0.5 and 2.0 in bivariate logistic regression were also included in the multivariate model. If 2 predictors showed a strong correlation with each other (0.7–1), then only one was included in the multivariate modelling. Age and sex were included regardless.

## Results

A total of 1024 children were identified by the key informants, 343(34%) children referred did not have CP while (388/731(53%) were confirmed to have CP at that point in time. The mean age of the children with CP was 9.2 years (SD) ±4.0). There were 229 (59%) males and 159 (41%) females. Carers reported seeking treatment for CP first in the hospital in 56.7%.

Ambulatory children (GMFCS I-III) made up 280/388 (72%) while GMFCS IV-V were 108(28%) of the children with CP. Spastic CP was the most common type (271/388,70%), and was bilateral in 163/271(60%) and unilateral in 108(40%).

### Comorbidities

Comorbidity distribution are shown in Table 1.

**Table 1:** Distribution of comorbidities and other variables in an unadjusted bivariate analysis showing predictors of school attendance (*n* = 388)

Type of comorbidity	N (%)	Crude OR	95% CI		*P* value
Feeding difficulties	334 (86)	0.06	0.01	0.3	< 0.0001
Learning difficulties	342 (88)	15.8	6.8	36.7	< 0.0001
Speech impairment	331 (85)	2.6	1.5	4.7	0.001
Swallowing difficulties	299 (77)	0.3	0.2	0.6	< 0.0001
Abnormal Behaviour (Total difficulties score)	231 (62)	1.2	0.8	1.9	0.355
Visual acuity impairment	209 (54)	6.3	3.9	10.1	< 0.0001
Communication difficulties (CFCS 4–5)	173 (45)	4.8	2.9	7.8	< 0.0001
Objective perceptual visual disorders	177 (46)	5.2	1.2	22.8	0.027
Epilepsy	130 (35)	3.0	1.8	4.9	< 0.0001
Hearing impairment	46 (12)	1.3	0.6	2.5	0.468
Malnutrition	200 (51)	1.8	1.2	2.8	0.005
GMFCS IV-V	108 (28)	16.1	6.3	40.8	< 0.0001
MACS 4–5	88 (23)	20.2	6.2	65.4	< 0.0001

{OR > 1 means significantly associated with poor school attendance, OR < 1 means significantly associated with better school attendance}

Subcategories of abnormal behaviours included; Difficulties getting along with other children 240(63%), reduced kind & helpful behaviour 252(67%), hyperactivity and inattention 162(43%) and abnormal conduct 233(62%). Neonatal seizures 108/388(28%) (OR 4.4, 95% CI 2.8–7.1; *p* < 0.001), were four times more likely in children with epilepsy. Irregular antiepileptic medications were used in 7/130(5.4%) children while others used none.

Malnutrition was seen in 200/388(51%) and was associated with MACS 4–5, 62/88(70%); (OR 2.8,95% CI: 1.7–4.7; *p* < 0.001), GMFCS IV-V, 83/108(77%) (OR 4.6; CI:2.8–7.7;7*p* < 0.01). Conversely a negative association was seen with both feeding, 161/334(48%) (OR 0.3; CI:0.2–0.7; *P* < 0.001) and swallowing difficulties 140/299(47%) (OR 0.4; CI:0.2–0.7; *p* < 0.001).

**Fig. 1:**
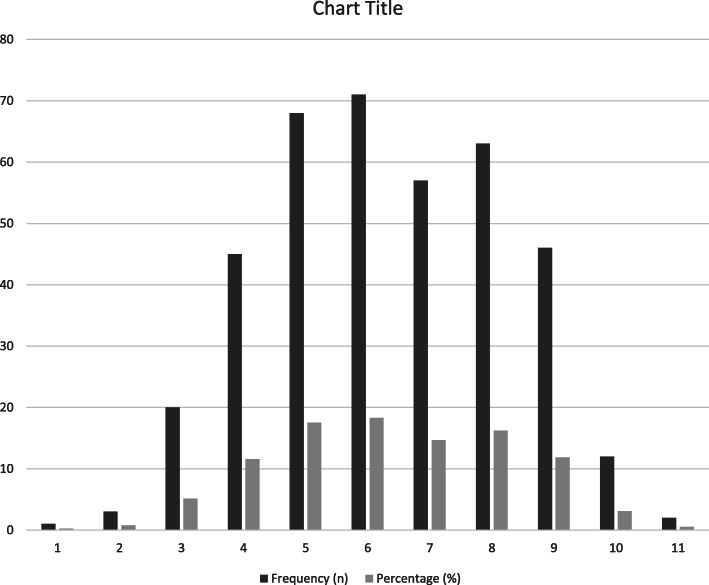
Number and frequency of comorbidities in children with CP (*n* = 388)

The comorbidity score showed a mean of 6·4 (SD 1·9; median 6; IQR 5,8), with the Kruskal-Wallis test showing a significant difference in the distribution of the co-morbidity scores between the CP clinical types ((X^2^ (4) = 10.921, p < 0·0275); Dystonic CP showed the highest number of co-morbidities; 7.4 (SD 1·8 Median 7.5, IQR 6,9). Children with more than 5 comorbidities accounted for 65% of children and at least 1 comorbidity was seen in every child (Fig. 1 and supplementary material 1).

### Schooling

All the children recruited were of school age, 115/388(30%) had never attended school, and 145 of the 273(53%) who started school dropped out; of the 128/388 (33%) in mainstream school, for 124/128 (97%) children, parents reported that the children were behind in academic performance. As at the time of recruitment, two thirds of children were not in school (260/388, 67%).

### Multivariate analysis

Of the reported bivariate predictors of school attendance only swallowing and feeding difficulties showed some correlation (*R* = 0.7) hence swallowing difficulties was deleted from the multivariate model.

Age and sex adjusted multivariate analysis is shown in, Table 2.

**Table 2:** Multivariate logistic regression analysis of comorbidities and other factors predicting poor school attendance in children with CP (*n* = 388)

Variables	Multivariate analysis			
Type of comorbidity	Adjusted OR	*P* value	95% CI	
Age > 9 years	0.3	0.001	0.2	0.6
GMFCS IV-V	6.9	0.002	2.0	24.0
MACS 4–5	4.7	0.049	1.0	22.2
Feeding difficulties	0.1	0.039	0.02	0.9
Learning difficulties	10.1	< 0.001	3.6	28.1
Visual acuity impairment	2.8	0.002	1.5	5.3
Epilepsy	2.3	0.009	1.2	4.3

## Discussion

This population based study on children with CP from Nigeria a LMIC study, suggests evidence on specific comorbidities and their negative impact on school attendance which were independent of CP severity.

Previous studies on CP in children from Nigeria and LMIC have mostly been from facility based samples rather than the community. There have recently been population-based studies from Bangladesh on prevalence and co-morbidity [24], using the same methodology and from Uganda, on prevalence only [17]. Review of these, has highlighted there is a need for further population-based studies from other LMIC to understand cultural and geographic differences in the burden of co-morbidities that has differed across regions [42]. .Furthermore, few studies in LMIC are available on children with CP from community-based studies in relation to participation in schooling and most information regarding schooling, have been based on hospital samples [5, 43].

Comparative studies have shown that the KIM can be used effectively to estimate a prevalence and identify associated comorbid conditions and predictors. For instance, a large sample of children with CP with physical impairment have been identified through the key informant method in Bangladesh [21, 24, 28]. However, the issues of stigma, difficulty with movement and poor expectations for treatment of the condition may have discouraged some parents from bringing their child for examinations [44].

All the comorbid conditions in our study occurred in higher frequency than are reported in studies from High Income Countries [45, 46]. In the Ugandan study, two comorbidities were reported (learning disability and epilepsy) in similarly high frequencies and this was from a hospital-based study where one might expect higher level of difficulties that had resulted in referral to hospital [5]. In comparison to Bangladesh [24], the proportion with various co-morbidities was similar for some e.g. hearing impairment and higher in others e.g. visual acuity impairment. Differences seen could well be related to different screening and assessment measures as well as reflecting population differences. Our study also included other areas of difficulty which can have a significant impact such as behaviour and malnutrition.

In our population, very few of the modifiable comorbidities, such as epilepsy, were receiving treatment. Feeding and swallowing difficulties were very common but were surprisingly associated with significant reduced likelihood of malnutrition compared with severe CP and manual ability 4–5, which both showed an increased likelihood for malnutrition. Similar studies [47, 48], have shown an association between feeding and swallowing problems and malnutrition [49]. ,What may appear as a discrepancy may be as a result of the cultural feeding norms where from the age of above 4 years in these communities, feeding is communal with all the children in the household feeding from the same plate together, with the older child expected also to assist the younger children to obtain food from the plate. Children with severe CP and manual disability, are most unlikely to compete with their normal peers for the food. Suggesting the aetiology of malnutrition may not only be as a result of the difficulties in swallowing or chewing in these communities in children above 4 years of age.

Similar to a population based study from Uganda [5], children with dystonia had the highest mean comorbidity score. This may reflect more global insult from underlying aetiology of CP e.g. neonatal encephalopathy in these children. Comorbidity is associated with worse health outcomes, more complex clinical management, and increased health care costs [50]. The relationship between the number and specific comorbidity per child and mortality in children with CP requires further investigation across LMIC.

The importance of participation in education by children with disability including CP has been reiterated by several organizations [6]. We found no child attending special education school in our study. A low prevalence was seen in another Nigerian hospital-based study where only 8% attended special education schools [43]. One of the major reasons identified by the earlier study for keeping the children away from school was fear of stigma and the assumption from family members that the children were not capable of learning [43]. It is possible that parents may have noticed some of the comorbidities but did not understand or assumed that they could not be addressed. The academic expectation of children with CP should be tailored and agreed with parents. Understanding the link between poor school performance and comorbidity would help towards more individualised child centred approaches of care. For example, the consequence of untreated epilepsy could result in deleterious cognitive and behavioural consequences [51], both of which could be ameliorated.

Significant determinants of poor school attendance in this population independent of the severity of CP based on ambulation and manual disability, are: epilepsy, learning difficulties and visual acuity impairment. Some of these have proven effective interventions when indicated [49], for example, the use of antiepileptic medication in some children and the use of spectacles in children with refractive errors and/or accommodative dysfunction which are known to be beneficial [52, 53]. Interventions may improve the quality of life, school participation, performance and favourable competition with their peers [54]. Apart from environmental and social interventions which addresses non-ambulation and manual ability, interventions to ameliorate comorbidities in children with CP towards improvement in schooling should be considered. Focus on the development of special education schools and complex facilities to improve capacity for clinical care, habilitation and education of children with more severe CP may be beneficial.

Children older than 9 years were seen to have a reduced likelihood of poor school attendance. There may be a link between mortality of children with CP and different age groups as well as between mortality and the development of adaptations in children with CP. These require further investigations in the implication for school attendance. A similar protective finding of significance was feeding difficulty; These was seen maybe because of the ongoing school feeding programme where parents who are aware their children’s malnourished state, but do not understand the cause of the malnutrition send their children to school to participate in the school feeding programme. More investigations would clarify reasons why this is seen.

### Limitations of the study

Recruitment using key informant methodology is not as rigorous as door to door population surveys but is more practical and cost effective and has been used for large and well analysed studies of CP in Bangladesh [21, 24]. Our KIM methodology has been based on the Bangladesh model however, it may have missed children resulting in selection bias. For instance, it may have been that children with more severe and stigmatising CP were not brought thus possibly underestimating the degree of co-morbidity in this population and hence the public health significance. Identification of comorbidities in some areas relied on parental report with possible recall bias however, there were additional extensive professional assessments used to determine comorbidity. Lastly, our choice of cut-off values affects the frequency of co-morbidities and also most likely their impact as predictors of the outcome variables. In spite of these limitations, the study probably reflects this country’s best case scenario [10], used a well-established sampling methodology, trained personnel as interviewers, international classifications, and it represents a large sample in a LMIC population.

## Conclusion

CP severity contributes significantly to poor school attendance, hence the social model of care in disability should continuously be strengthened. However, the majority of school-aged children with CP in this large population-based study in Southern Nigeria showed a high prevalence of multiple, untreated co-morbidities highlighting CP in this population as a multimorbid condition which may be contributing adversely to school attendance. Some of these co-morbidities that present as barriers are modifiable and if properly managed, may have the potential to have positive impacts on school attendance.

United Nations Sustainable goal four is for quality education and over half of the children who are not enrolled in school globally are in sub Saharan Africa and Nigeria is by far the largest country in sub Saharan Africa. The sustainable goals emphasise the principle of not leaving anyone behind (universal coverage) and this study highlights that comorbidity is likely to be a major impediment to school attendance for children with CP in low and middle income countries which requires urgent attention.

## Supplementary Information

**Additional file 1 Supplementary material 1.** Number and frequency of comorbidities in children with CP (*n* = 388)

## Data Availability

The datasets used and/or analysed during the current study are available from the corresponding author on reasonable request.

## Abbreviations

CP: Cerebral palsy
GMFCS: Gross motor function classification scale
MACS: Manual ability classification scale
CFCS: Communication function classification scale
KIM: Key informant methodology
LMIC: Lower middle income country
