# Supplementary material for: Pattern of comorbidities in school-aged children with cerebral palsy in Cross River State, Nigeria
Source: BMC Pediatr. 2021 Apr 8;21:165. doi: 10.1186/s12887-021-02637-9 (PMC8028192; doi:10.1186/s12887-021-02637-9)
Supplement: Supplementary file 1 — Additional file 1 Supplementary material 1. Number and frequency of comorbidities in children with CP (n = 388) [file 12887_2021_2637_MOESM1_ESM.docx]

Supplementary material 1: Number and frequency of comorbidities in children with CP (n=388)

| No of comorbidities | Frequency (n) | Percentage (%) |
| --- | --- | --- |
| 1 | 1 | 0.26 |
| 2 | 3 | 0.77 |
| 3 | 20 | 5.15 |
| 4 | 45 | 11.6 |
| 5 | 68 | 17.53 |
| 6 | 71 | 18.3 |
| 7 | 57 | 14.69 |
| 8 | 63 | 16.24 |
| 9 | 46 | 11.86 |
| 10 | 12 | 3.09 |
| 11 | 2 | 0.52 |
